# Interactive effect of sleep duration and sleep quality on risk of stroke: An 8-year follow-up study in China

**DOI:** 10.1038/s41598-020-65611-y

**Published:** 2020-05-26

**Authors:** Ailing Ji, Heqing Lou, Peian Lou, Chunrong Xu, Pan Zhang, Cheng Qiao, Qing Yang

**Affiliations:** 1 Department of Neurology, Xuzhou Third People’s Hospital, 131 Huancheng Road, Xuzhou 221000 Jiangsu, China; 2 Department of Control and Prevention of Chronic Non-communicable Diseases, Xuzhou Center for Disease Control and Prevention, 142 West Erhuan Road, Xuzhou 221000 Jiangsu, China

**Keywords:** Stroke, Risk factors

## Abstract

Inappropriate sleep duration and poor sleep quality are associated with risk of stroke, but their interactive effect on stroke is unknown. We explored the interactive effect of sleep quality and duration on stroke risk. A prospective cohort study was conducted with 41,786 adults. Sleep quality was assessed using the Pittsburgh Sleep Quality Index. Sleep duration was measured by average hours of sleep per night. Cox regression models were used to calculate the association of sleep duration and quality with stroke. The delta method and a non-conditional logistic regression model were used and the relative excess risk due to interaction (RERI), the attributable proportion (AP), and the synergy index (S) were calculated. Compared with sleep duration 6–8 h/day, the risk ratio of stroke was 1.63 (1.23–2.11) times for sleep duration <6 h/day and 1.40 (1.08–1.75) times for >8 h/day. The stroke risk ratio was 2.37 (1.52–3.41) times in subjects with poor sleep quality compared with those with good sleep quality. Women who slept <6 h/day had higher stroke risk than men who slept <6 h/day. Men who slept >8 h/day had higher stroke risk than women who slept >8 h/day. Men with poor sleep quality had higher stroke risk than women with poor sleep quality. Stroke was associated with short/long sleep duration and poor sleep quality in subjects aged >46 years, compared with those aged 18–45 years. Stroke occurred more frequently in subjects with poor sleep quality combined with short sleep duration (odds ratio: 6.75; 95% confidence interval (CI): 2.45–14.12). RERI, AP, and S values (and their 95% CIs) were 5.54 (3.75–8.12), 0.72 (0.56–0.80), and 5.69 (4.23–9.90) for the poor sleep quality interact with short sleep duration. In persons with poor sleep quality accompanied by long sleep duration, RERI, AP, and S (95% CI) were 1.12 (1.01–1.27), 0.35 (0.26–0.51), and 2.05 (1.57–2.96), respectively. Subjective sleep disturbances are related with risk of stroke in Chinese adults. There are additive interactions between short/long sleep duration and poor sleep quality that affect risk of stroke.

## Introduction

As the top cause of death and disability and the second leading cause of years of potential life lost, stroke killed over 6 million deaths in 2015 worldwide, and the number will be doubled by 2030, with over 200 million disability-adjusted life years each year^1,2^. The burden is the biggest in China: 1,114.8/100,000 of the age-standardized prevalence rate in 2013^3^ and annual increase of 11.9% for the first-ever stroke morbidity during 1992-2015^4^. With respect to the traditional risk factors of stroke in China, hypertension, smoking, alcohol use, diabetes, and hypercholesterolemia are prevalent and could also be modified^3,5^. Some studies have shown that stroke is associated with poor sleep quality and sleep duration^6–8^, while others have insistent results^9,10^, which might partly due to different races^11^. Therefore, sleep quality in relation with stroke should be further investigated to determine whether stroke prevention programs should incorporate efforts to reduce sleep disturbances in different races.

Sleep disturbances and insufficient sleep quantity are very common in Chinese adults^12,13^. However, few studies report the relationship between sleep quality and stroke^14,15^. Therefore, identifying and describing the relationship, and implementing early intervention measures to are extremely important in modifying the risk factor and in further slowing the progression of the disease in China. To this end, the current study aims to examine the effects and the interactive effect of sleep quality and sleep duration on stroke, respectively, in Chinese adults.

## Methods

### Study protocol and population

The baseline survey with a sample selected from all of the eleven regions of Xuzhou city, Jiangsu province, China, was conducted in, during May to November 2009. A two-stage sampling probability proportional to size (PPS) was used, and each region was considered a stratum, yielding a total of 11 strata. Five subdistricts/townships in urban/rural areas were firstly selected randomly from each stratum using PPS based on the population data. Then five communities/villages were randomly selected from each subdistrict/township using PPS and a total of 275 villages or communities, as clusters, were selected. In each selected village or community, a list of households containing adults aged ≥18 years and living their current residence for at least 5 years was generated. Finally, a Kish selection table was used to select one person from each household. The exclusion criteria included: (1) previous diagnosis of neuropathy, psychosis, or difficulty in communication, or (2) the floating population or temporary residents, or (3) obstructive sleep apnea. At least 13,500 people should be recruited assuming an estimation incidence of stroke of 2.0%^15^ with 90% power, α level of 0.05, and allowing for a dropout rate of 15%. Overall 41,786 permanent residents aged ≥18 years participated in the survey, with a response rate of 91.6% (41,786/45,618). All participants were rechecked from May to November 2017. A total of 672 participants had stroke, and 1,585 participants were excluded owing to missing information about sleep duration or sleep quality; diagnosis of neuropathy or psychosis; use of any kind of psychotropic medication; severe heart, liver, or kidney disease, and/or cancer, producing a total of 39,533 participants in this prospective observational study. The interval of follow-up duration was the time between the baseline assessment and the diagnosis of stroke, death, or the date on which the 2017 questionnaire was finished. Those who developed strokes or died from strokes in the follow-up, and those who fulfilled the final research, were included in the present study. The main reasons for participant loss were death, migration, or other personal reasons at follow-up. All participants were of Han nationality.

### Key measures

Sleep quality and disturbance over a 1-month length was estimated by a Chinese version of the Pittsburgh Sleep Quality Index (PSQI)^16^. This version has seven components and a total score from 0 to 21 points with reliability coefficient of 0.82–0.83 and acceptable test–retest reliability of 0.77–0.85^17^. PSQI score of ≤5 and >5 was correspondingly differentiated as good and poor sleep quality, respectively. The diagnostic sensitivity and specificity in differentiating poor from good sleepers were 89.6% and 86.5%, respectively. Sleep quantity was calculated on self-reported average total hours of per night sleep in the previous year, and was categorized as <6, 6–8, and >8 h per night, following previous studies^6,7,12^.

In the follow-up, data on strokes were collected using self-reports and medical record questionnaires. Self-reported stroke should meet the criteria of an acute disorder of focal areas in the brain continuing for ≥24 h caused by intracranial hemorrhage or ischemia^18^ with a diagnosis of computed tomography and magnetic resonance imaging scans. By definition, asymptomatic stroke and transient ischemic attack were excluded.

### Variables

Participants were interviewed face-to-face and privately by trained interviewers using a standard questionnaire that assessed age, gender, marriage, occupation, educational level, cigarette use, alcohol use, physical activity, history of disease (type 2 diabetes mellitus (T2DM), obstructive sleep apnea, dyslipidemia, hypertension, heart disease, and cancer), and family history of disease (T2DM, hypertension, and stroke). Occupation was defined as manual, non-manual, unemployed, and retired. Educational level was classified as below high school, high school, and above high school level. Marriage was categorized as single, divorced, widowed, or separated; living conditions were sorted out as living alone or with someone else. Those who have smoked at least 100 cigarettes over the lifetime were defined as smokers. A drinker was defined as the consumption of no less than 30 g alcohol per week lasting 1 year or more. Physical activity was defined as joining moderate or vigorous activity for at least 30 min per day no less than 3 days a week. Weight in kilograms divided by height in meters squared was defined as body mass index (BMI) with categorized as underweight (≤18.5 kg/m^2^), normal weight (18.5–24.0 kg/m^2^), and overweight/obese (≥24.0 kg/m^2^); Women with waist circumference of >85 cm and men with that of >90 cm were defined as abdominal obesity^19^. T2DM was defined as fasting blood glucose of ≥7.0 mmol/L, or the use of any antidiabetic drugs, or self-reported history of T2DM^20^. Hypertension was characterized as systolic blood pressure ≥140 mmHg and/or diastolic blood pressure ≥90 mmHg, or the use of any antihypertensive drugs, or self-reported history of hypertension^21^. Dyslipidemia was defined as any lipid-lowering medication use or self-reported history of dyslipidemia. History of cardiovascular disease was defined as previous diagnosed ischemic heart disease and/or cerebrovascular events. A score of ≥3 on the STOP-Bang questionnaire indicated the presence of obstructive sleep apnea^22^.

### Statistical analysis

Epidata 3.1 was used to build a database. Data were input separately by two people and a conformance test was performed. All statistical analyses were conducted by SPSS 16.0 (SPSS Inc., Chicago, USA).

Categorical variables were presented as frequencies and proportions or means (±standard deviation) and medians (interquartile range). Baseline characteristics of participants and non-participants in the follow-up examination were compared using the chi-square test, Student’s t-test, and the Mann–Whitney U test, as appropriate. The Mann–Whitney U test and Fisher’s exact test were used to compare the characteristics and sleep duration/qualities of participants who developed stroke with those who did not develop stroke. Risk ratios (RRs) and corresponding 95% confidence intervals (CI) were calculated using Cox regression models with reference to the risk of 7–8 h/day of sleep duration or good sleep quality, adjusting for age, gender, marital status, current employment status, educational level, BMI, cigarette use, alcohol intake, physical activity, DM2, fasting plasma sugar, lipids, dyslipidemia, hypertension, heart disease, family history of DM2, family history of hypertension, and family history of stroke. The observed prevalence of stroke was plotted and stratified by sleep quality and sleep duration. RRs and 95% CIs were also calculated for sleep duration and sleep quality in relation with age, adjusted by potential confounding factors, (gender, marital status, current employment status, educational level, BMI, cigarette use, alcohol intake, physical activity, DM2, fasting plasma sugar, lipids, dyslipidemia, hypertension, heart disease, family history of DM2, family history of hypertension, family history of stroke, and sleep quality and duration).

The Excel table compiled by Anderss *et al*.^23^ was introduced into the database using the delta method and relevant indicators of interaction were computed. The value obtained from the logistic regression model was taken as the estimated additive interaction between sleep quality and sleep duration. The following measures of interaction were examined to estimate biological interactions: the relative excess risk due to interaction (RERI), the attributable proportion due to interaction (AP), and the synergy index (S)^24,25^. The RERI is the excess risk attributed to the interaction relative to the risk without exposure. AP refers to the attributable proportion of disease caused by the interaction in subjects with both exposures. S is the excess risk from both exposures when there is a biological interaction relative to the risk from both exposures without interaction. In the absence of additive interactions, RERI and AP are equal to 0^25^. Indicative biological interactions would be considered when RERI > 0, AP > 0, or S > 1. A *P* value <0.05 (two-tailed) was considered statistically significant.

## Results

### General characteristics of participants

A total of 39,533 participants (males, 48.1%) who did not have stroke at baseline and 27,712 out of them completed the follow-up in 2017, of whom 617 developed stroke The median follow-up duration was 7 years. At baseline, the mean age and the average sleep duration per night were 45.7 ± 15.1 years and 7.16 ± 1.07 h in 2009, respectively. The non-responder group was older (50.7 ± 16.8 *vs* 44.6 ± 15.1 years, *P* < 0.001), with more short- sleepers [(2,098/11,821) *vs*. **(**5,368/27,712), *P* < 0.01], more long-sleepers [(2,021/11,821) *vs* **(**5,232/27,712), *P* < 0.01], and more individuals with poor sleep quality [(1,053/11,821) *vs* **(**1,989/27,712), *P* < 0.01]. There were no significant between-group differences in other risk factors. With respective of the 27,712 participants, those with stroke tended to be older, smokers and drinkers and have a higher BMI. There were no significant gender differences in the other risk factors (Table 1).

**Table 1:** Patients’ baseline characteristics in 2009 by presence of stroke (N = 27,712).

	Total	Stroke		*P* value
		Yes (n = 617)	No (n = 27095)	
**Sex**				
Male	13247	334 (54.1)	12913 (47.7)	
Female	14465	283 (45.9)	14182 (52.3)	0.002
**Age** (**years)**	**45.7** ± **15.1**	**61.0** ± **15.5**	44.6 ± 15.2	<0.001
**Occupational**				
**Manual**	21789	432 (70.0)	21357 (78.8)	
**Non-manual**	1703	37 (6.0)	1666 (6.1)	
**Unemployed**	1761	33 (5.3)	1728 (6.4)	<0.001
**Retired**	2459	115 (18.1)	2344 (8.7)	
**Education level**				
**Below high school**	11457	280 (45.4)	11177 (41.3)	0.043
**high school**	14339	306 (49.6)	14033 (51.8)	
**Above high school**	1916	31 (5.0)	1885 (7.0)	
Not married	2490	23 (3.7)	2467 (9.1)	<0.001
Living alone	7141	103 (16.7)	7038 (26.0)	<0.001
**Smoker**	5621	204 (33.1)	5417 (20.0)	<0.001
**alcohol use**	4402	133 (21.6)	4269 (15.8)	<0.001
**Regular exercise**	6699	141 (22.9)	6558 (24.2)	0.467
**Family history of DM2**	735	13 (2.1)	722 (2.7)	0.468
**Family history of stroke**	578	21 (3.4)	557 (2.1)	0.030
**Family history of hypertension**	2097	53 (8.6)	2044 (7.5)	0.371
BMI (≥24 kg/m^2^)	12374	397 (64.3)	11977 (44.2)	<0.001
**Hypertension**	6524	403 (65.3)	6121 (22.6)	<0.001
**DM2**	1943	103 (16.7)	1840 (6.8)	<0.001
**FPG** (mmol/L)	5.6 ± 1.2	5.6 ± 1.2	6.4 ± 1.5	<0.001
Dyslipidemia	3714	111 (18.0)	3603 (13.3)	<0.001
TC (mmol/L)	4.67 ± 1.12	4.67 ± 1.12	4.72 ± 1.13	0.277
HDL-Cl (mmol/L)	1.40 ± 0.32	1.40 ± 0.32	1.37 ± 0.33	0.025
LDL-C (mmol/L)	2.59 ± 0.90	2.60 ± 0.90	2.56 ± 0.92	0.285
TG (mmol/L)	1.58 (1.11, 2.07)	1.57 (1.11, 2.07)	1.61 (1.12, 2.15)	0.245
<**6**	5386	172 (27.9)	5196 (19.2)	<0.001
>**8**	5232	143 (23.2)	5089 (18.8)	0.007
**Sleep quality**				
**Good**	8050	145 (23.5)	7905 (29.2)	0.002
**Poor**	1898	87 (14.1)	1811 (6.7)	<0.001

Unless indicated otherwise, data are means ± standard deviations, medians (interquartile ranges) or frequencies (%). *P* values are from the Mann–Whitney U test (continuous variables) or Fisher’s exact test (categorical variables). Dyslipidemia: LDL-C ≥ 2.6 mmol/L, HDL-C < 1.0 mmol/L, TG ≥ 2.3 mmol/L, and/or concurrent use of lipid-regulating drugs.

BMI: body mass index; DM2: type 2 diabetes mellitus; FPG: fasting plasma sugar; TC: total cholesterol; HDL-C: high-density lipoprotein cholesterol; LDL-C: low-density lipoprotein cholesterol; TG: triglycerides.

### Sleep quality and sleep duration in relation with stroke

The cumulative incidence of stroke was 2.22% in participants who completed the study. Individuals with short sleep duration (<6 h/day) significantly increased risk of stroke compared with those with sleep duration 6–8 h/day (for all, RR: 1.63, 95% CI: 1.23–2.11, *P* < 0.001; for men, RR: 1.45, 95% CI: 1.19–1.73, *P* < 0.001; for women, RR: 1.77, (95% CI: 1.41–2.37, *P* < 0.00), after adjusting for confounding factors. Women had a slightly stronger relationship than men. Individuals with long sleep duration also increased risk of stroke compared with those with sleep duration 6–8 h (for all, RR: 1.40, 95% CI: 1.08–1.75, *P* < 0.01; for men, RR: 1.48, 95% CI: 1.02–1.57, *P* < 0.01; for women, RR; 1.23, 95% CI: 1.12–1.79, *P* < 0.01), after adjusting for confounding factors (Table 2). males had a slightly stronger relationship than females. The population-attributable risk of short and long sleep duration on risk of stroke was 0.35 and 0.23, respectively. Subjects with poor sleep quality greatly raised risk of stroke compared with those with good sleep quality (RR: 2.37, 95% CI: 1.52–3.41, *P* < 0.001). The subgroup analysis classified by gender found a similar association between men and women. The RRs were 2.65 (95% CI: 1.72–4.57, *P* < 0.01) for men and 2.13 (95% CI: 1.45–2.77, *P* < 0.01) for women after adjustment, the association was stronger in males than that in females. The population-attributable risk of poor sleep quality on risk of stroke was 0.39. There was no significant association between good sleep quality and stroke with normal sleep quality as reference (RR: 1.10, 95% CI: 0.95–1.40, *P* = 0.336) (see Table 2). Subgroup analysis classified by age showed only a significant association between sleep duration, sleep quality and stroke for participants aged >46 years (Tables 3 and 4).

**Table 2:** Risk ratios for the association between sleep quality, sleep duration, and stroke (N = 27,712).

Variables		Stroke	No stroke	RR (95%CI)	P
Sleep quality	Good	145 (1.8%)	7895	1	
	Common	385 (2.2%)	17391	1.10 (0.95–1.40)	0.336
	Poor	87 (4.6%)	1809	2.37 (1.52–3.41)	<0.001
Sleep duration	6–8 h	302 (1.8%)	16823	1	
	<6 h	172 (3.2%)	5190	1.63 (1.23–2.11)	<0.001
	>8 h	143 (2.7%)	5082	1.40 (1.08–1.75)	<0.01

Adjusted for age, gender, marital status, current employment status, educational level, body mass index, cigarette use, alcohol intake, physical activity, type 2 diabetes mellitus, dyslipidemia, hypertension, heart disease, family history of type 2 diabetes mellitus, family history of hypertension, family history of stroke, fasting plasma sugar, and lipids. RR: risk ratio; CI: confidence interval.

**Table 3:** Risk ratios (95% confidence intervals) for stroke according to sleep quality by age.

ages	sleep quality	Stroke		RR (95%CI)	P
		Yes	No		
18–35	Good	10	1549	1	
	Common	8	1544	0.72 (0.03–1.65)	0.36
35–45	Good	30	1850	1	
	Common	35	1805	1.05 (0.53–2.55)	0.62
	Poor	16	775	1.15 (0.38–6.84)	0.4
46–55	Good	22	1438	1	
	Common	92	4319	1.27 (0.81–2.29)	0.24
	Poor	24	527	2.58 (1.52–7.87)	0.007
55–65	Good	42	1931	1	
	Common	164	5628	1.24 (0.96–2.42)	0.07
	Poor	34	512	2.64 (1.15–4.75)	<0.001
65–80	Good	39	1629	1	
	Common	83	3431	0.96 (0.80–2.85)	0.74
	Poor	18	127	5.39 (2.15–8.42)	<0.001

Adjusted for gender, marital status, current employment status, educational level, body mass index, cigarette use, alcohol intake, physical activity, type 2 diabetes mellitus, dyslipidemia, hypertension, heart disease, family history of type 2 diabetes mellitus, family history of hypertension, family history of stroke, fasting plasma sugar, and lipids.

RR: risk ratio; CI: confidence interval.

**Table 4:** Risk ratios (95% confidence intervals) for stroke according to sleep duration by age.

ages	sleep duration	Stroke		RR (95%CI)	P
		Yes	No		
18–35	6–8 h	11	2009	1	
	<6 h	4	565	1.15 (0.94–1.82)	0.46
	>8 h	3	519	1.01 (0.21–2.02)	0.45
35–45	6–8 h	51	2867	1	
	<6 h	18	741	1.24 (1.01–3.05)	0.002
	>8 h	12	822	0.69 (0.45–1.54)	0.57
46–55	6–8 h	79	4050		
	<6 h	29	1099	1.25 (1.01–3.04)	<0.001
	>8 h	30	1165	1.24 (1.01–2.85)	<0.001
55–65	6–8 h	99	4631	1	
	<6 h	73	1591	1.91 (1.12–3.63)	<0.001
	>8 h	68	1849	1.49 (1.16–2.67)	<0.001
65–80	6–8 h	62	3266	1	
	<6 h	47	1194	1.81 (1.22–3.76)	<0.001
	>8 h	31	727	1.92 (1.23–5.00)	<0.001

Adjusted for, gender, marital status, current employment status, educational level, body mass index, cigarette use, alcohol intake, physical activity, type 2 diabetes mellitus, dyslipidemia, hypertension, heart disease, family history of type 2 diabetes mellitus, family history of hypertension, family history of stroke, fasting plasma sugar, and lipids.

RR: risk ratio; CI: confidence interval.

### Sleep quality interacted with sleep duration on risk of stroke

The results from the multiple logistic regression models were showed in Table 5. Individuals with poor sleep quality combined short sleep duration significantly increased risk of stroke compared with those with good sleep quality combined sleep duration 6–8 h (RR: 1.39, 95% CI: 1.13–1.70 and RR: 1.35, 95% CI: 1.09–1.67, respectively, *P* < 0.001).

**Table 5:** Risk ratios for the association between sleep quality and stroke by sleep duration.

Sleep duration	Sleep quality	stroke	No stroke	RR (95%CI)	P
6–8 h	Good	105	6307	1	
	Common	168	9409	1.00 (0.92–1.30)	0.210
	Poor	29	1071	1.39 (1.13–1.70)	<0.001
<6 h	Good	26	1006	1.35 (1.09–1.67)	<0.001
	Common	113	3935	1.55 (1.24–1.83)	<0.001
	Poor	32	249	6.75 (2.45–14.12)	<0.001
>8 h	Good	14	582	1.23 (0. 95–1.34)	0.257
	Common	104	4011	1.37 (1.10–1.73)	<0.001
	Poor	26	489	2.77 (1.41–7.99)	<0.001

Adjusted for age, gender, marital status, current employment status, educational level, body mass index, cigarette use, alcohol intake, physical activity, type 2 diabetes mellitus, dyslipidemia, hypertension, heart disease, family history of type 2 diabetes mellitus, family history of hypertension, family history of stroke, fasting plasma sugar, and lipids. RR: risk ratio; CI: confidence interval.

The risk of stroke was greatest in those with poor sleep quality combined short sleep duration (RR: 6.75, 95% CI: 2.45–14.12, *P* < 0.001). In addition, subjects with long sleep duration and good sleep quality had no increased risk of stroke compared with those who had good sleep quality and sleep duration of 6–8 h (RR: 1.23, 95% CI: 0.95–1.34, *P* = 0. 257). Those with both poor sleep quality and long sleep duration had higher risk of being stroke (RR: 2.77, 95% CI: 1.41–7.99, *P* < 0.001) compared with those with sleep duration of 6–8 h.

### Sensitivity analysis

There was a strong additive interaction between sleep quality and sleep duration on stroke, with 72% of stroke events attributed to the interaction between poor sleep quality and short sleep duration and 35% attributed to the interaction between poor sleep quality and long sleep duration(Table 6).

**Table 6:** Estimates of the biological interaction between sleep quality and sleep duration for stroke.

Measures of biological interaction	Estimate (95% CI)
**Poor sleep quality versus sleep duration <6 h**	
RERI	5.54 (3.75–8.12)
AP	0.72 (0.56–0.80)
S	5.69 (4.23–9.90)
**Poor sleep quality versus sleep duration >8 h**	
RERI	1.12 (1.01–1.27)
AP	0.35 (0.26–0.51)
S	2.05 (1.57–2.96)

Reference group is good sleep quality and 6–8 h sleep duration. RERI: relative excess risk due to interaction; AP: attributable proportion due to interaction; S: synergy index; CI: confidence interval.

## Discussion

Our study showed that poor sleep quality and sleep duration (<6 h/d or >8 h/d) increased the risk of being stroke independent of the potential confounders in a Chinese population. This association was only found in participants aged >45 years, not in those aged 18–45 years. Sex differences showed that women had a slightly stronger relationship between sleep duration of <6 h/d and stroke than men, while men had a slightly stronger relationship between. In addition, there was also an interactive effect of long sleep duration and poor sleep quality on stroke. The findings suggest that sleep quality and quantity should be included in the evaluation of stroke risks and development of prevention methods and interventions.

Many studies indicated that both short and long sleep duration increased risk of stroke^7,26–29^, consistent with our study. Eguchi *et al*. conducted a multivariable Cox regression analysis with 1,268 hypertensive patients and found that sleep duration less than 7.5 h/d was independently associated with stroke risk (hazard ratio = 2.21; *P* = 0.003)^26^. One systematic review analyzed with 474,684 participants from 15 studies (24 cohort samples) and suggested that short sleep duration was positively related with risk of developing stroke (relative risk: 1.15, 95% CI: 1.00–1.31, *P* = 0.047)^27^. A 8-year cohort study including 23,620 participants found that risk of stroke increased 2.06 times (95% CI: 1.18–3.59) in those with sleep duration <6 h/d compared with those with duration 7-8 h/d^28^. A meta-analysis by Ge and Guo with 12 cohort studies reported a hazard ratio for stroke risk of 1.13 (95% CI: 1.02–1.25) in individuals having short sleep duration^7^. Results from a meta-analysis included six cross-sectional studies showed an pooled odds ratio of 1.71 (95% CI: 1.39–2.02) for individuals with short sleep duration developing stroke^7^. Li *et al*. reported a pooled relative risks by 7% per hour of sleep reduction for stroke events were among individuals having sleep duration <7 h/d^29^. Furthermore, our findings showed a U- shaped association between sleep duration and risk of stroke, consistent with previous report^30^. However, there was difference by sex and age. The risk of being stroke seemed slightly higher in female with short sleep duration, and in male with long sleep duration. Both short and long sleep duration were associated with stroke in participants aged 46-80 years, while this association was not found younger ones, consistent with a 10-year cohort study report^31^. The results suggest that flexibility to insufficient sleep or vulnerability to prolonged sleep as risk factors for stroke may differ by age.

Our study also found risk of stroke in relation with poor sleep quality, and men had a slightly stronger relationship than women. Zhang *et al*. Reported^14^ an odds ratio of 1.811 (95% CI: 1.116–2.939) for being stroke among poor-quality sleepers aged 18–45 years compared with good-quality sleepers. Conversely we found the association only occurring in the age over 46 years, which may be partly due to higher risk of carotid intima-media thickness in relation with poor sleep quality in stroke-free individuals aged ≥40 years^32^.

Short sleep duration may increase cortisol levels via activation of sympathetic activity and hypothalamic–pituitary–adrenal axis^33,34^, which can further result in hypertension and harm brain tissue, and accelerate the development of stroke. Short sleep duration also results in insulin resistance, which alters vascular endothelial function, causes abnormal fibrinolysis and systemic inflammation, and elevates high-sensitivity C-reactive protein levelsand hypercoagulability^35–38^, which tends to promote atherosclerosis and subsequent stroke^35,39,40^.

Poor sleep quality is related to impaired glucose tolerance and insulin sensitivity, and increased interleukin 6 level, and plasma concentration^41–43^, which increases the risk of stroke.

Consistent with previous reports, the current study showed an association between stroke and long sleep duration. Cappuccio and colleagues systematically analyzed 15 studies including 474,684 participants and had a result of positive relationship between long sleep durationand risk of stroke with odds ratio of 1.65 (95% CI: 1.45–1.87, *P* < 0.0001)^26^. A 10-year cohort study including 7,844 adults showed a greater risk of stroke in persons with sleep duration >8 h per night than those with duration of 6–8 h per night (relative risk: 1.5, 95% CI: 1.1–2.0)^31^ A 7.5-year prospective study found a positive association between long sleep duration and ischemic stroke in 93,175 women aged 50–79 years^44^. Similar results were also reported by a prospective study including 9,692 stroke-free participants aged 42–81 years long sleep duration was significantly associated with an increased risk of stroke with a hazard ratio of 1.46(95% CI: 1.08–1.98) for being stroke in long-time sleepers^45^. Meta-analyzed results from the 12 cohort and 6 cross-sectional studies showed t a hazard ratio of 1·40(95% CI: 1.16–1.64) and odds ratio of 2.12 (95% CI: 1.51–2.73), respectively, for risk of stroke in individuals with long sleep duration^7^. Another meta-analysis of 11 studies showed a pooled relative risk of increased 17% per hour of sleep increment in those having sleep duration >7 h/d compared with those with 7 h/d^29^.

Sleep duration more than 8 hour is reported association with elevated blood C-reactive protein, interleukin 6, fibrinogen, and total leukocyte numbers, and decreased albumin levels, compared with sleep duration of 6–8 h^46–49^. Increases in inflammatory markers can damage blood sugar stability and β-cell function, leading to insulin resistance and the development of stroke. Taken together, the evidence shows that long sleep duration increases inflammatory cytokines and insulin resistance, and poor sleep quality also increases inflammatory cytokines and decreases insulin sensitivity; these factors all increase stroke risk.

Some main traditional modifiable risk factors are prevalent in China. For example, the incidence of hypertension is as high as 32.5%^50^, the incidence of diabetes is 11.6%^51^, and the prevalence of smoking is 28.3%^52^. As a relatively new modifiable risk factor, the prevalence of sleep disorders is 41.5%^33^. Therefore, the government and general practitioners should seriously consider both traditional and new modifiable risk factors in the development of stroke prevention methods and interventions. Given the difficulties of screening and treating stroke patients, recommendations to improve sleep quality and sleep duration are inexpensive and practical means of decreasing risk of stroke.

Our study has some limitations. Firstly, information on sleep quality and duration was self-reported, information bias may be inevitable. Secondly, we did not include clinical diagnoses for sleep disorders. Thirdly, other confounders associated with sleep disorders, such as depression and general health^53^, were not measured. Fourthly, our findings were derived from Chinese subjects which may not be generalized to other ethnic populations. Fifthly, lost-to-follow-up participants were older, and had poor sleep quality and short or long sleep duration, which may reduce the strength of the associations. Sixth, recreational drug use was not collected, which might be small affect on the relationship of sleep quality and stroke.

In summary, poor sleep quality and short/long sleep duration increase the risk of being stroke. Moreover, there is an interactive effect of poor sleep quality and short sleep duration on increased risk of stroke. As inappropriate sleep duration and poor sleep quality are common in Chinese adults, appropriate identification and treatment of sleep disturbances is very important to prevent the development of stroke. In the further, We would analyze the association between the specific sleep disorders, sleep patterns, environmental, behavioral and medical comorbid risk factors and stroke, and added the evidence of the relationship between sleep and stroke.

### Ethics approval and consent to participate

The study protocol was approved by the Xuzhou Center for Disease Control and Prevention, and conducted in accordance with the principles of the Declaration of Helsinki (1975, revised 2000). All participants provided written informed consent.

## Data Availability

All data relevant to the given manuscript have been stored in a separate file that can be made freely available to external investigators upon request.
